# Investigating Changes in Land Use Cover and Associated Environmental Parameters in Taihu Lake in Recent Decades Using Remote Sensing and Geochemistry

**DOI:** 10.1371/journal.pone.0120319

**Published:** 2015-04-21

**Authors:** Changchun Huang, Hao Yang, Yunmei Li, Jun Zou, YiMing Zhang, Xia Chen, Yin Mi, Mingli Zhang

**Affiliations:** 1 Jiangsu Center for Collaborative Innovation in Geographical Information Resource Development and Application, Nanjing Normal University, Nanjing, Jiangsu, China; 2 School of geography science, Nanjing Normal University, Nanjing, Jiangsu, China; 3 Jiangsu Provincial Key Laboratory of Materials Cycling and Pollution Control, Nanjing Normal University, Nanjing, Jiangsu, China; 4 Key Laboratory of Virtual Geographic Environment (Nanjing Normal University), Ministry of Education, Nanjing, Jiangsu, China; Tennessee State University, UNITED STATES

## Abstract

Humans have had a significant impact on the terrestrial pedosphere through activities such as agriculture and urbanization. The effects of human activities on land use and the related environmental changes were investigated through point and areal studies surrounding Meiliang Bay, which is an open area of extreme eutrophication in Taihu Lake, China. This study used remote sensing and environmental-tracer profiles [total nitrogen (TN), total phosphorus (TP), total organic carbon (TOC), grain size, and geochemical parameters] to determine the causes of changes in land use and the associated environmental parameters. The results of LUCCs (Land use/cover changes) indicate that over the past three decades, total farmland decreased by 862.49 km^2^, with an annual decrement rate of 28.75 km^2^/year, and total urbanized land increased by 859.71 km^2^, with an annual growth rate of 28.66 km^2^/year. The geochemical results indicate that the trophic state of Taihu Lake was persistently intensifying and that the TN, TP, and TOC concentrations increased twofold, threefold, and twofold, respectively, from 1949 to 2010. The sources of TN, TP, and TOC were highly similar after 1975. However, before 1974, TN and TP originated from different sources than TOC. The grassland and woodland around the lake retain nutrients and sand from the land of study area. The increase in urbanized land and tertiary industries significantly increased the sediment concentrations of TN, TP, and TOC after 1980.

## Introduction

The impact of human activities on the terrestrial pedosphere has increased considerably over the past two centuries [1, 2]. Worldwide, approximately 4.7 million km^2^ of grassland and 6 million km^2^ of woodland have been converted to agricultural and urbanized land since 1850 [3, 4]. Land provides essential resources for life as well as production for humans; however, land use and cover changes (LUCCs) have become a primary driver of biodiversity loss, climate change, species invasion, and environmental change [5–8]. LUCCs also alter the land’s net radiation and sensible and latent heat ratios by modifying the reflectance of the land surface [5, 9]. Decreases in humidity and precipitation due to increased radiation reduce the regeneration ability of forests [10–13]. Soil and water quality are significantly affected by LUCCs as well because different land use types have different sewage purification and production capacities, water consumption rates, and storage capacities. The impacts of LUCCs on the soil and water are manifested in soil erosion, degeneration of soil physicochemical properties, water cycles, and pollution [14–18]. LUCCs alter the nutrients and carbon cycles in the soil and water [19–21]. Thus, natural vegetation and soil systems have considerably greater nutrient cycling abilities than manmade agricultural systems [22]. Forests typically have considerably smaller nitrogen and phosphorus losses than other land types [23, 24]. As the ultimate store of sand and nutrients lost from land surface, the sedimentary core contains large information of environmental changes [25–28]. The records of sedimentary core can animatedly reproduce the human activity, hypoxia, eutrophication of Lake and environmental change [27, 29–31]. Consequently, the records of environmental change surround the sedimentary core can be found in the sediment.

In economically developed regions, large quantities of nutrients are released into aquatic ecosystems from the surrounding land [32]. However, the high frequency of LUCCs in these regions requires high-time-resolution monitoring tools. Remote sensing is a powerful and essential tool for monitoring LUCCs because it facilitates observations over a larger area and at a higher frequency than ground-based observations [33–35]. Satellite images can also be used to track historical LUCC information (the Landsat satellite data can be traced back to 1972) [8]. Lake sediments record information on the evolution of the environment [36–38]. Satellite imagery and sediment core data were determined to be superior tools for studying the impact of LUCCs on the environment.

The goal of this study is to explore the effects of human activities and LUCCs on environmental changes by 1) analyzing changes in land use and environmental parameters around Meiliang Bay (Taihu Lake, China) using Landsat imagery (TM/ETM+) and sediment core data (total organic carbon (TOC), total nitrogen (TN), and total phosphorus (TP)) and 2) quantifying the spatial and temporal patterns of land use changes in this area between 1980 and 2010.

## Material and Methods

### 2.1 Ethics statement

The field investigation site is located at the south of Wuxi city (population: 6.95 million). No specific protected water, land areas or plant/animal species were issued from either the government or relevant authorities. No specific permits were required for the described field studies, and the work did not involve any endangered or protected species.

### 2.2 Study area

**Fig 1:**
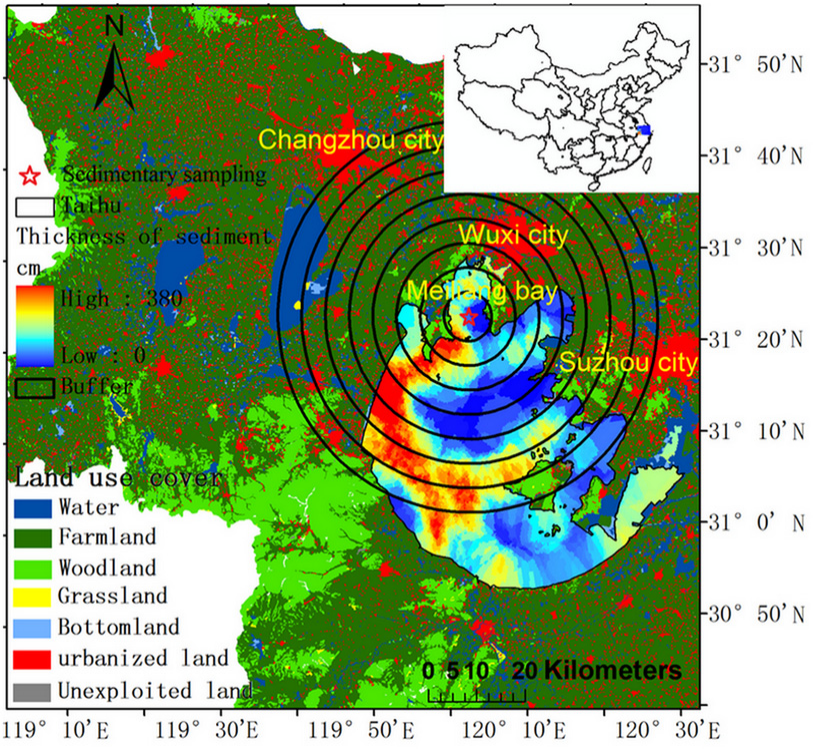
Study area. The auxiliary chart in the top right corner shows the location of the study area in China. The background image of the land surrounding Taihu Lake shows the land use cover in 2000. The background of Taihu Lake provides the distribution of the sedimentary thickness. The black point is the monthly water quality monitoring site. The red pentagram is the sediment sampling point. The buffer of sediment includes 8 regions, with the radius of each buffer ranging from 5 to 40km at 5km intervals.

Taihu Lake is a well-known shallow inland lake in the Yangtze River delta; Fig 1 presents the location of the study area in China. The key issues in Taihu Lake are hyper-eutrophication and algal blooms [39, 40]. The total nitrogen concentration increased from 0.9 to 2.18 mg/L between 1981 and 2009. The total phosphorus concentration increased from 0.04 to 0.075 mg/L between 1987 and 2009. The TN and TP concentrations in Meiliang Bay are considerably higher than those in Taihu Lake. For instance, the mean TP concentration in Meiliang Bay (0.13mg/L) is higher than that in Lake Taihu (0.07 mg/L), and the mean TN concentration in Meiliang Bay (2.35 mg/L) is higher than that in Lake Taihu (2.21 mg/L) [41].

### 2.3 Data and methods

#### I. data collection

Four sedimentary cores were collected from Taihu Lake in October 2012. A gravity-type columnar sediment sampler was used to collect the columnar sediment. The sediment samples were frozen at—50°C for 48 h, cut into 0.5 cm-thick slices (depth of sediment is 30 cm), and dried in a lyophilizer. The TP and TN concentrations of the dry sediment samples were measured with a UV-3600 spectrophotometer (Shimadzu Corp., Japan), the TOC concentration of the samples was measured with a TOC analyzer (Shimadzu Corp., Japan), and sedimentary grain size was measured by Mastersizer (Mastersizer 2000, Britain). The ^210^Pb, ^226^Ra and ^137^Cs isotopes were measured with a Gamma Spectrometer (ORTEC., USA).

Landsat (TM/ETM+) images with a spatial resolution of 30 m were used to obtain land use cover data from 1980 to 2010. The time phases of land use cover data include 1980, 1995, 2000, 2005, 2008, and 2010. The Landsat images for each time phase were geo-referenced according to the match up geographic coordinates (geometric correction). All geo-referenced images for each time phase were corrected by quick atmospheric correction module before classification. The classification method is a combination method of artificial visual interpretation and computer intelligence interactive interpretation (SVM: support vector machine). The Landsat images were interpretated by computer intelligence interactive interpretation firstly, and then artificial visual interpretation and correction were used to correct the classification results from computer intelligence interactive interpretation. The finally classification accuracy was guaranteed with 90%.

The weather and statistical data for the city of Wuxi were chosen for this study because most of the study area is in this city (the coverage percentages of Wuxi, Suzhou, and Changzhou are 70%, 20%, and 10%, respectively). Information on the population, domestic water usage, and industrial water usage was obtained from the Wuxi statistical yearbook. The weather data (precipitation) for Wuxi from 1955 to 2010 were downloaded from the China Meteorological Data Sharing Service System (http://cdc.cma.gov.cn/).

#### II. Methods

Among of four sediment cores, only the sediment core in Meiliang Bay satisfies the condition of geochronology by ^210^Pb radiometric technique (constant rate of supply, CRS) [42, 43]. The sediment core was dated using the activity of unsupported ^210^Pb (^210^Pb_ex_) radiometric technique based on the method suggested by Mizugakia [42]. The ^210^Pb_ex_ was calculated by subtract the ^226^Ra from ^210^Pb. The time estimated by the ^210^Pb_ex_ radiometric technique was calibrated by ^137^Cs, which has significant peaks in the years 1963 and 1986. Specific historical data in hydrology and meteorology (flooding and strong rainfall) were used to correct the geochronology estimated by ^210^Pb_ex_ as well. The geochronology of this sediment core is from 1902 to 2010.

The study area (Meiliang Bay) is an open basin with densely covered river network. Thus it’s hard to discuss the influence of land use cover change on the environmental changing due to the fragmentized drainage basin. Enlightened by land-use regression model [44–46], we chose this sediment core and established 8 buffers surrounding this sediment core (Fig 1) to analyze the effect of LUCCs to environmental changes based on the spatial correlation of geography.

The deposition rate (cm/a) is calculated from the depth and geochronology.

deposition rate=(Z2-Z1)/[geochronology(Z1)-geochronology(Z2)]

where, Z1 and Z2 is the sedimentary depth, geochronology(Z1) and geochronology(Z2) are the date for the depth of Z1 and Z2.

## Results

### 3.1 Land use and cover change from 1980 to 2010 around the sediment sampling sites

Table 1 presents the land use change in a 40 km-radius buffer from 1980 to 2010. Total urbanized land increased by 859.71 km^2^, with an annual growth rate of 28.66 km^2^/year over the past three decades. The increased urbanized land (867.77 km^2^) originated from woodland (15.83 km^2^), grassland (1.69 km^2^), water (15.23 km^2^), unexploited land (0.90 km^2^), and farmland (834.12 km^2^). The decreased urbanized land (8.06 km^2^) was converted to woodland (0.7 km^2^), water (1.09 km^2^), unexploited (0.1 km^2^), and farmland (6.71 km^2^). In contrast to urbanized land, total farmland decreased by 862.49 km^2^, with an annual reduction rate of 28.75 km^2^/year. The decreased farmland (872.75 km^2^) was mainly converted to urbanized land (834.12 km^2^), water (24.09 km^2^), and woodland (14.23 km^2^). Farmland only increased by 10.25 km^2^. The increase in farmland (10.25 km^2^) resulted from the conversion of woodland (1.79 km^2^), grassland (0.1 km^2^), water (2.19 km^2^), and urbanized land (6.17 km^2^). Woodland and grassland decreased by an average of 4.98 and 2.99 km^2^/year, respectively. Water and unexploited land increased by an average of 9.56 and 1.19 km^2^/year, respectively. New urbanized land is mainly converted from farmland. The increase in unexploited land is mainly due to the urbanization process.

**Table 1:** Land use change in the 40 km buffer between 1980 and 2010 (km^2^).

		Woodland	Grassland	Water	Urbanized	Unexploited	Farm
1980–1995	Increase	2.49	0.10	2.39	226.45	0.00	27.67
	Decrease	1.59	0.20	0.70	26.38	0.00	230.23
	Change	0.90	-0.10	1.69	200.07	0.00	-202.56
1995–2000	Increase	1.29	0.10	2.69	127.21	0.10	20.60
	Decrease	13.14	1.39	1.49	17.42	0.00	118.55
	Change	-11.84	-1.29	1.19	109.79	0.10	-97.94
2000–2005	Increase	9.36	0.00	14.83	176.38	0.00	10.05
	Decrease	0.90	0.00	9.16	10.45	0.00	190.12
	Change	8.46	0.00	5.67	165.93	0.00	-180.06
2005–2008	Increase	0.00	0.40	1.29	248.25	0.10	0.00
	Decrease	1.49	0.00	5.67	0.00	0.00	242.87
	Change	-1.49	0.40	-4.38	248.25	0.10	-242.87
2008–2010	Increase	7.27	0.00	11.35	187.83	2.29	43.30
	Decrease	8.26	1.85	5.97	52.16	1.29	182.35
	Change	-1.00	-1.85	5.38	135.67	1.00	-139.05
Total Change		-4.98	-2.84	9.56	859.71	1.19	-862.49

**Fig 2:**
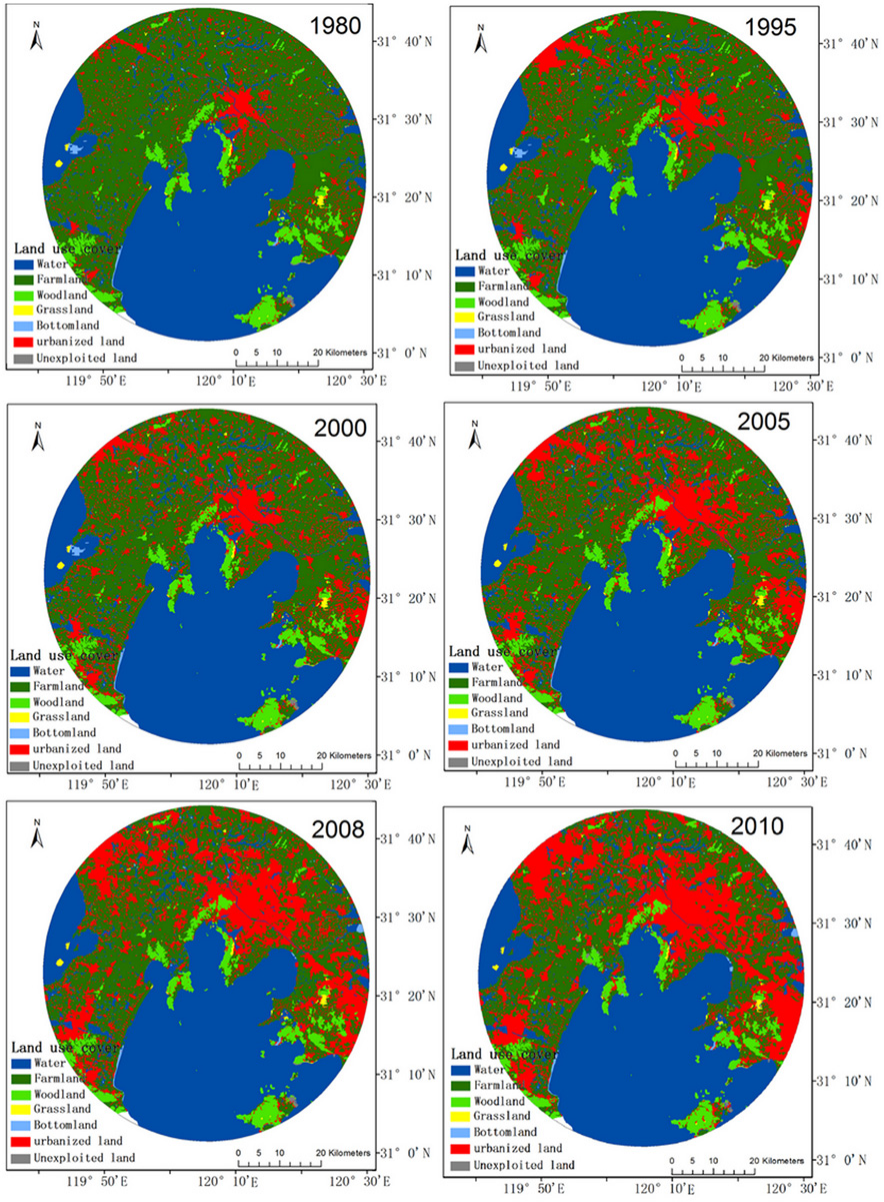
Distribution of land use cover in the 40 km radius from 1980 to 2010.

The distribution of land use also shows that the urbanization is a process of farmland occupation (Fig 2). The pace of urbanization in this study area has been rapid, especially after 2005. Thus, the LUCCs mainly indicate a decrease in farmland and increase in urbanized land as well as a decrease in grassland within buffers of 30, 35, and 40 km.

### 3.2 Distribution of total organic carbon, total nitrogen, and total nitrogen in the sedimentary cores

**Fig 3:**
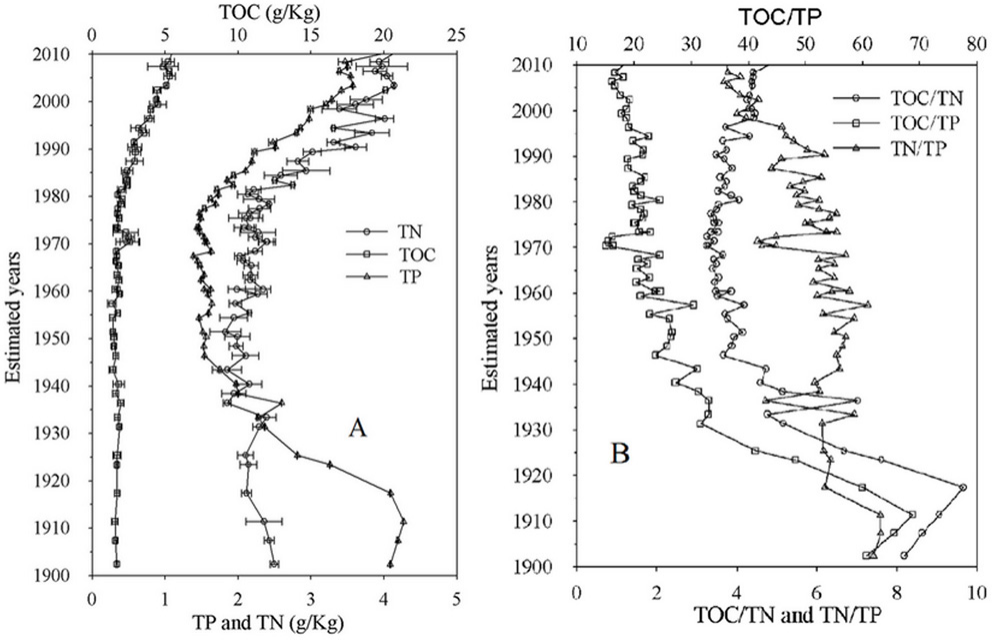
Distribution of soil constituents in the sediment core. Chart A shows the distribution of TN, TP, and TOC in the different layers of the sediment, which was dated by ^210^Pb. Chart B shows the distribution of the ratios of TN, TP, and TOC in the different layers of the sediment.

Sedimentary cores contain a considerable amount of information on environmental changes. Fig 3A presents the distribution of TN, TP, and TOC over the study period. TP exhibited an increasing trend after 1975, and the growth rate of TP increased from 1975 to 2010. A peak value of TP was observed around 1972. TP was nearly constant before 1970. Sedimentary TP increased threefold from 1900 to 2010 (from 0.4 to 1.2 μg/mg). TN exhibited a decreasing trend between 1900 and 1945, a fluctuating trend between 1945 and 1975, and an increasing trend after 1975. Sedimentary TN decreased twofold from 1900 to 1945 (from 4.1 to 1.97 μg/mg) and increased twofold from 1975 to 2010 (from 2.1 to 4.3 μg/mg). TOC exhibited an increasing trend with fluctuations between 1900 and 2010. Sedimentary TOC increased approximately threefold from 1900 to 2010 (from 7.1 to 21.6 μg/mg).

The ratio of TOC and TP (TOC/TP) exhibited a decreasing trend after 1910 and an increasing trend before 1910. TOC/TP decreased fourfold from 1910 to 2010 (from 68.7 to 17.8). The ratio of TOC and TN (TOC/TN) decreased approximately threefold from 1900 to 1979 (from 9.6 to 3.4) and increased approximately twofold from 1980 to 2010 (from 3.4 to 5.3). The ratio of TN and TP (TN/TP) exhibited a fluctuating trend before 1990 and a decreasing trend after 1990.

### 3.3 Distribution of sedimentary grain size and deposition rate

**Fig 4:**
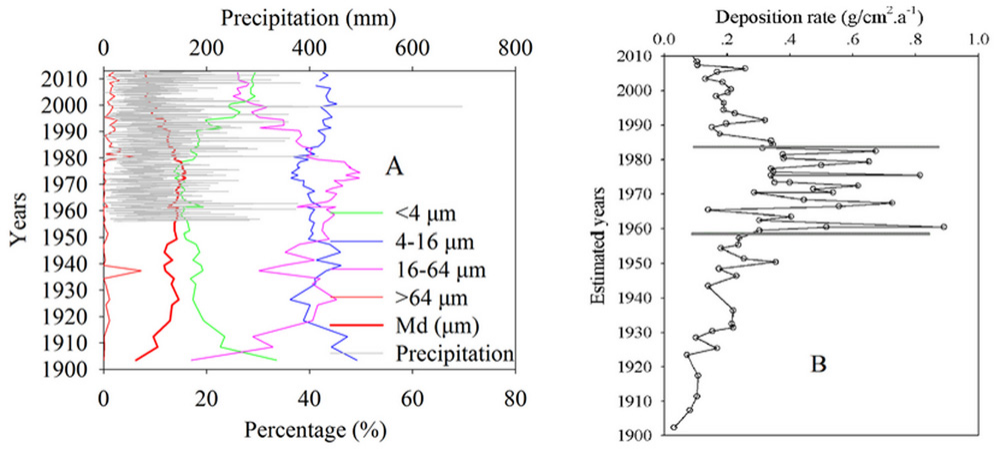
Chart A is the distribution of sedimentary grain size for the different size ranges, the bold red line is the median diameter. Chart B is the distribution of the deposition rate calculated by sedimentary quality depth, density, and date.

Fig 4A presents the sedimentary grain size, which was separated into clay (<4μm), fine-grained sand (4–16μm), coarse-grained sand (16–64μm), and sand (>64μm) according to the Udden-Wentworth scale. The percentage of clay decreased from 32.7% to 15.9% between 1900 and 1948, increased from 17.2% to 29.5% between 1976 and 2010, and maintained a stable trend between 1949 and 1979 (mean value of 15.5%). The percentage of fine-grained sand decreased slightly from 43.6% to 36.5% between 1900 and 1972 and slightly increased from 36.5% to 42.6% between 1972 and 2010. The mean percentage of fine-grained sand from 1900 to 2010 was 41.2%. The percentage of coarse-grained sand increased threefold (from 17.1% to 49.8%) from 1900 to 1973 and decreased from 49.8% to 25.9% between 1973 and 2010. The percentage of sand was less than 1% from 1900 to 1979, except in 1931, when a catastrophic flood increased the percentage of sand to 7.3%. The percentage of sand exceeded 1% from 1979 to 2010, with a mean value of 1.6%. The maximum percentage of sand in that time period occurred in 1981 (5.9%), which was caused by another catastrophic flood. The sedimentary deposition rate exhibited an increasing trend from 1900 to 1960 (from 0.02 to 0.89 g/cm^2^a^-1^), reached a maximum range between 1960 and 1980 (mean value is 0.48 g/cm^2^a^-1^), and then decreased to 0.08 g/cm^2^a^-1^ in 2011.

## Discussion

### 4.1 Influence of land use and cover changes on environmental changes

**Fig 5:**
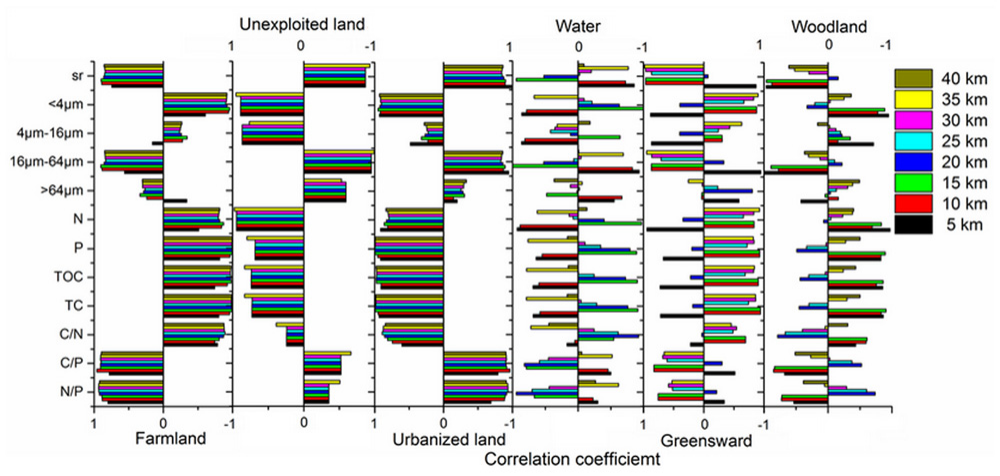
Pearson correlation coefficients between LUCCs and the environmental indices. Six years were included in the correlation analyses, namely, 1980, 1995, 2000, 2005, 2008, and 2010, and sr is the median diameter.

LUCCs change not only the landscapes in which human live but also the climate, biodiversity, and the eco-environment [47–49]. The LUCCs have a significant effect on environmental changes from the correlation analysis between LUCCs and environmental indices (Fig 5). TN, TP and TOC have highly negative correlations with farmland for all buffers, indicating that more TN, TP and TOC will input to the lake during the transformation process of farmland to other land use types (Table 1 and Fig 3A). Unexploited and urbanized lands may be the main contributors to TN, TP, and TOC for all buffers because they displayed high positive correlations. The correlations of woodland and grassland with TN, TP, and TOC rely heavily on the buffer radius. Grassland is negatively correlated with TN, TP, and TOC within the 5 km buffer and positively correlated with these parameters within the 10–35 km buffers. Woodland is positively correlated with TN, TP, and TOC within the 5–15 km buffers and has almost no correlation with TN, TP, and TOC within the 20–40 km buffers. The possible explanations for these observations are that the grassland nearby the sedimentary core plays a retaining role for the nutrients, but the grassland far from the sedimentary core is a source of nutrients for the artificial cultivation. The woodland in the buffers is mainly landscaped woodland, which with low capacity of soil and water conservation relative to the wildwood [18, 50]. Sometimes, in order to keep the growth of landscape trees, lots of fertilizer may be applied.

Clay (<4μm) mainly originates from unexploited and urbanized land, whereas fine-grained sand (4–16μm) mainly originates from unexploited land and coarse-grained sand (16–64μm) mainly originates from farmland. Grassland and woodland can play positive roles in soil conservation.

The TN output from farmland was considerably higher than that from urbanized land, and the output difference of TN between farmland and urbanized land increased over time (agriculture ranged from 43,037 t to 59,674 t and urbanized land ranged from 32,492 t to 36,954 t in 1994 and 1998) [41]. The TP output from farmland was considerably lower than that from urbanized land (agriculture ranged from 1,385 t to 4,625 t and urbanized land ranged from 3,985 t to 7,742 t in 1994 and 1998) [41]. The decrease in farmland should reduce the concentration of TN in sediment because farmland can produce large quantities of TN from underutilized fertilizer. The decreasing fertilizer use from 2000 to 2012 (114311 ton decreased to 59738 ton) (from agricultural statistics) should reduce the concentration of TN in sediment further. However, this trend was not observed (Fig 3).

### 4.2 Effect of changes in demographic structure on environmental changes

**Fig 6:**
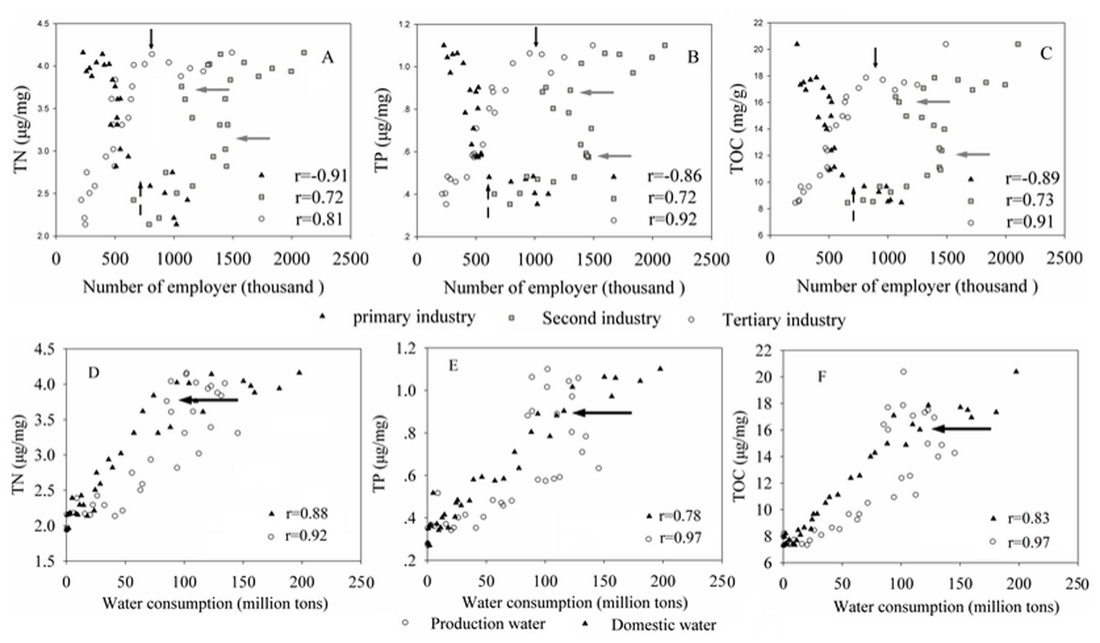
Relationships of environmental indices, population and water. The environmental indices include TN, TP, and TOC, and the population includes employees in the primary, secondary, and tertiary industries, the water includes the production and domestic water.

As the economy develops and farmland is lost, farmers become migrant workers. Thus, the population, influenced by the LUCCs, can change the various industry clusters (primary, second, and tertiary industries), which is considered another influencing factor to the TN, TP, and TOC. Fig 6 presents the TN, TP and TOC changes with changes of population in the primary, secondary, and tertiary industries from 1978 to 2010. TN, TP, and TOC increased as the farmers gradually became migrant workers and other types of workers, particularly after 1985 (the dotted arrow points to the sampling location in Fig 6A–6C). TN, TP, and TOC increased significantly with the growth of the tertiary industry population before 2002 (black bold arrow points to the sampling location in Fig 6A–6C). The influence of secondary industry on the concentrations of TN, TP, and TOC exhibited an increasing trend with two inflection points in the years 1991 and 2002 (two gray arrows point to the sampling location in Fig 6A–6C). This may be due to the following two reasons. First, a large group of workers was laid-off during the period of the early 1990s to 2000. Second, as part of a water diversion project, water was diverted from the Yangtze River to Taihu Lake to wash the lake.

With the development of industry and urbanization, the amount of pollution entering the rivers and lake has increased and is associated with the rapid increase in water requirement and wastewater discharge. The concentrations of TN, TP, and TOC grew significantly with the increase in domestic water usage. However, the growth rate of TN and TOC decreased after 2002. The influence of water production on TN, TP, and TOC was clear before 2002, but this influence weakened after 2002 (black arrow points to the sampling in Fig 6D–6F).This trend indicates that domestic wastewater may be the main source of TN, TP, and TOC in the study area. The government should prioritize the treatment of domestic wastewater when the industrial wastewater is well under control.

### 4.3 Effect of human activities on environmental changes

The median diameter of the sediment can be used to indicate the human activities, precipitation, and hydrodynamic energy during the deposition process [51]. The low changing rate of the Taihu Lake area indicates low hydrodynamic energy [52]. Human activities and precipitation are the main influencing factors for the distribution of the median diameter in Meiliang Bay. The distribution of the median diameter should be divided into two parts, namely, before and after the completion of hydraulic engineering surrounding Taihu Lake, because the hydraulic engineering has significantly affected the distribution of the median diameter (Fig 4A bold red line and gray line). The hydraulic engineering projects in the Taihu Basin area were completed during the 1990s. The median diameter exhibited an increasing trend from 1900 to 1930 (from 6.32 to 13.18μm) before decreasing to 11.51 μm in 1945, possibly due to the war with Japan and Chinese Civil War. The median diameter increased steadily from 1949 to 1978 with a mean value of 14.59μm, possibly due to advancements from the Cultural Revolution. The median diameter then decreased continuously after 1978, possibly due to the implementation of reform and open policy and the recession of agricultural activities.

TOC/TN is a good indicator of the sources of sedimentary organic matter [53, 54]. The decomposition of organic matter increases the nitrogen and phosphorus contents and decreases the carbon content [55, 56]. Organic matter that can break down easily (e.g., phytoplankton) has a low TOC/TN ratio. Organic matter that cannot break down easily (e.g., terrestrial higher plants) has a high TOC/TN ratio and is highly resistant to degradation [57, 58]. The TOC/TN ratio in this study indicated that the sedimentary organic material in Meiliang Bay is produced by both planktonic and terrestrial organic matter. The terrestrial organic matter in this study mainly originates from agriculture (e.g., rice, legumes and wheat cultivation, excreta of livestock). The decrease in TOC/TN before 1950 may indicate a decrease in human activities, such as the abandonment of agriculture. The steady trend of TOC/TN from 1951 to 1979 may indicate that the human activities were relatively stable. The increase in TOC/TN since 1979 may indicate an intensification of human activities, such as the urbanization process (Fig 3B).

Rainfall, vegetation, and other influencing factors affect the deposition rate in inland lakes [59]. However, several studies have shown that deposition rates significantly increased in response to the soil erosion induced by intensive agricultural and urbanized processes [60–63]. The increase in deposition rates from 1900 to 1960 (from 0.02 to 0.89 g/cm^2^a^-1^) may demonstrate the reconstruction of the nation after a long war. The maximum range in the deposition rates from 1960 to 1980 may indicate intensive agriculture and construction without improved hydraulic engineering. The decrease in deposition rates after 1980 may indicate a decrease in agricultural activities and improvements in hydraulic engineering (Fig 4B). The increased deposition rates also allow additional organic matter to be degraded by anoxic processes because the exposure time of organic matter to dissolved oxygen in the water column is reduced (the low TOC and TOC/TN are marked in Fig 3, and the high deposition rates are marked in Fig 4B).

### 4.4 Homology source analyses of total organic carbon, total nitrogen, and total nitrogen

**Fig 7:**
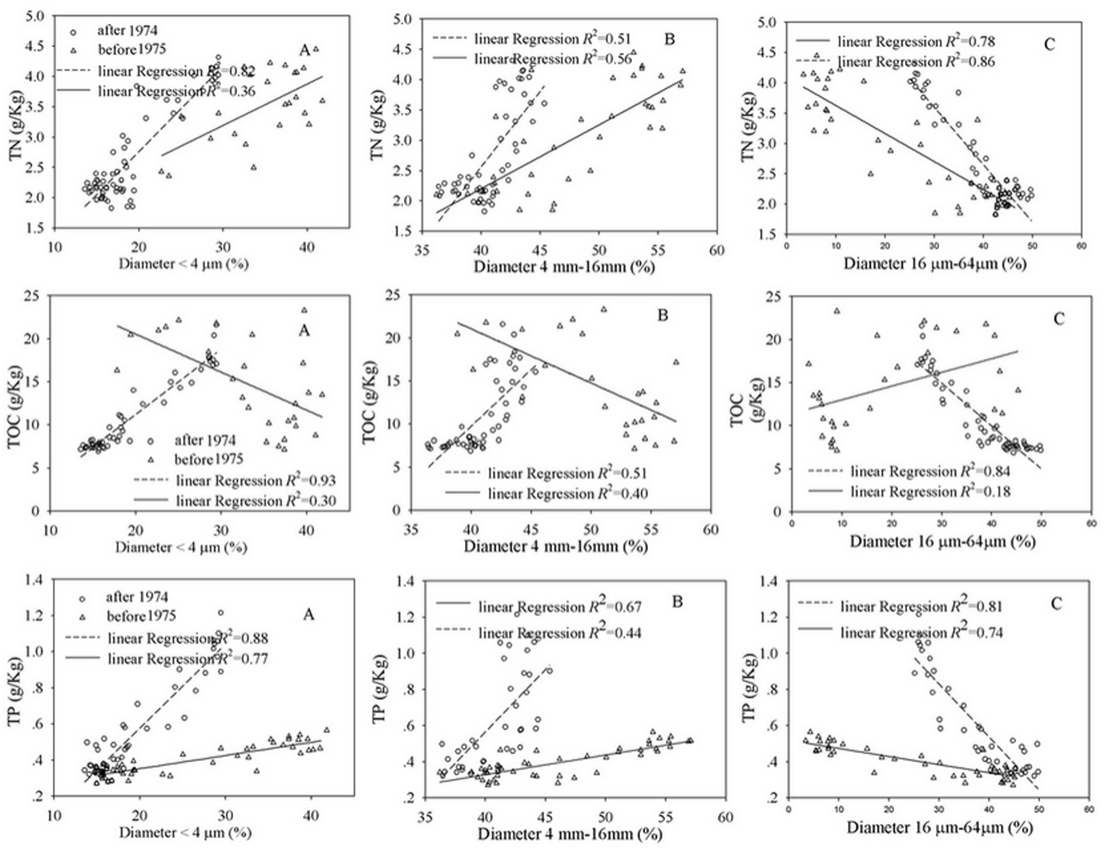
Relationship between grain size and environmental indices. The environmental indices include TN, TP, and TOC, and the grain size includes clay (<4μm), fine-grained sand (4–16μm), and coarse-grained sand (16–64μm).

The concentrations of TN, TP, and TOC increase with decreasing grain size because organic matter adsorbs onto mineral surfaces and has a high correlation with clay (<4μm) and fine-grained sediment (4–16μm) (Fig 7). The relationship between grain size and the concentrations of TN, TP, and TOC should be separated into two phases, namely, the period before 1974 and the period after 1975. These two phases correspond to the two periods during the development of China before and after the “reform and opening-up” policy was implemented. The concentrations of TN, TP, and TOC exhibited high linear correlations with clay and fine-grained sand (Fig 7).The TN and TP exhibited high positive correlations with the clay, indicating that the dynamic carrier of TN and TP is mainly clay. However, the carrying rates (the slopes of linear regression) are different in the two periods. The carrying rates of TN and TP by clay after 1974 were considerably higher than those before 1974. The TOC had a high positive correlation with clay (r = 0.93) after 1975 and almost no correlation with clay before 1974 (r = 0.30), suggesting that the TN, TP, and TOC in clay surrounding the Meiliang Bay significantly increased after 1975. TN, TP, and TOC had high negative correlations with the coarse-grained sand, and these correlations should also be separated into phases, namely, after 1975 and before 1974.

High correlation coefficients were observed between TN, TP, and TOC after 1975 (the correlation coefficient between TN and TP was 0.93; the correlation coefficient between TN and TOC was 0.96; and the correlation coefficient between TP and TOC was 0.98),and TN, TP and TOC had almost no correlation with each other before 1974 (the correlation coefficient between TN and TP was 0.73; the correlation coefficient between TN and TOC was -0.32; and the correlation coefficient between TP and TOC was 0.04).These correlation analyses indicate that TN, TP, and TOC originated from the same sources after 1975. The intense human activities in the area are likely the main source of TN, TP, and TOC, and the amount of TN, TP, and TOC originating from natural sources (e.g., the decomposition of phytoplankton, terrestrial organic matter and benthic invertebrates) was relatively smaller after 1975. TN and TOC had different sources before 1974, as did TP and TOC. The source of TP was not completely consistent with that of TN, indicating that TN, TP, and TOC originate from both natural sources and human activities, but natural sources may be more dominant than human activities in the future.

## Conclusions

The concentrations of TN, TP, and TOC in the sediment increased more than twofold from 1949 to 2010. The trophic state of Meiliang Bay showed a continuous increasing trend. The impact of LUCCs on environmental changes was significant. Most farmland was converted to urbanized land. The changes in demographic structure induced by LUCCs led to large discharges of TN, TP, and TOC into the water. The intense human activities in the area were likely the main source of TN, TP, and TOC, and the amount of TN, TP, and TOC originating from natural sources (e.g., the decomposition of phytoplankton, terrestrial organic matter and benthic invertebrates) was relatively smaller after 1975. TN, TP, and TOC originated from both natural sources and human activities, but natural sources may be more dominant than human activities in the future.
